# Is Atorvastatin Associated with New Onset Diabetes or Deterioration of Glycemic Control? Systematic Review Using Data from 1.9 Million Patients

**DOI:** 10.1155/2018/8380192

**Published:** 2018-10-22

**Authors:** Angeliki M. Angelidi, Emelina Stambolliu, Konstantina I. Adamopoulou, Antonis A. Kousoulis

**Affiliations:** ^1^Society of Junior Doctors, Athens, Greece; ^2^Hypertension Center STRIDE-7, Third University Department of Medicine, Sotiria Hospital, Athens, Greece; ^3^Faculty of Epidemiology and Population Health, London School of Hygiene & Tropical Medicine, London WC1E 7HT, UK

## Abstract

**Background:**

Current evidence indicates that statins increase the risk of new onset diabetes mellitus (NOD) and also deteriorate the glycemic control in patients with known diabetes mellitus (DM) after high-dose statin therapy.

**Aims:**

The aim of this review was to explore the effect of atorvastatin in causing NOD or deteriorating glycemic control in patients with DM.

**Methods:**

Two independent reviewers conducted the literature search, through PubMed database searching for articles published in English until April 2015, and only primary studies were included.

**Results:**

Of the 919 articles identified in our original search, 33 met the criteria for this review encompassing 1,951,113 participants. Twenty articles examined dysregulation of DM due to atorvastatin. Half of them showed that there was no significant change in glycemic control in patients treated with atorvastatin. Other studies showed that fasting plasma glucose and HbA1c levels were increased by atorvastatin. Thirteen articles examined if atorvastatin causes NOD. The majority of these articles showed that patients who used atorvastatin had a higher dose-dependent risk of developing NOD.

**Conclusion:**

This systematic review suggests that there is an association between atorvastatin treatment and NOD. Moreover, it showed that atorvastatin in high dose causes worsening of the glycemic control in patients with DM.

## 1. Introduction

Dyslipidemia is a primary well-established independent risk factor for cardiovascular disease [1]. An effective treatment, the 3-hydroxy-3-methylglutaryl coenzyme A (HMG-CoA) reductase inhibitors (also known as statins) are proven to lower low-density lipoprotein (LDL) cholesterol levels in patients with hypercholesterolemia [2].

Multiple prospective studies have showed the cardioprotective and antioxidant effects of statins, which have widely and for many decades been used for that purpose [3, 4]. LDL-cholesterol levels remain the principal target for lipid modification and statin therapy as the main treatment of achieving LDL goal attainment. The beneficial effect of statins in both primary and secondary prevention of cardiovascular events by lowering LDL-cholesterol concentrations has been documented among patients with or without diabetes [5, 6].

Diabetes mellitus is a growing public health problem that is approaching epidemic proportions globally, and it is also related with increased cardiovascular risk. In adults aged over 40 with diabetes mellitus, according to the American Diabetes Association (ADA) [7] and ACC/AHA guidelines [8], statin treatment is recommended, while it should also be considered for those less than 40 years old based on their risk profile.

Statin therapy is associated with significant reduction in cardiovascular endpoints; however, concerns have been raised over the use of statins and an increased risk of diabetes. Several statins are now available, with different potencies and drug interactions such as atorvastatin, pitavastatin, simvastatin, and rosuvastatin. However, their influence on insulin levels and insulin resistance has not been clarified yet. There are some theories suggesting a potential risk of developing new onset diabetes mellitus (NOD) [9] or a risk of deteriorating the glycemic control in patients with diabetes after high-dose statin therapy [10]. This risk is seemingly elevated with the use of atorvastatin [11]. Therefore, many clinical trials [12–14] have investigated the possible association between atorvastatin and new onset diabetes or dysregulation of already existing diabetes as well as the underlying mechanisms. It has also been reported that some groups with special characteristics, such as postmenopausal women [15] and renal allograft recipients [16], are in particular danger. On the other hand, few studies have demonstrated that atorvastatin did not worsen insulin sensitivity in patients with diabetes [17, 18], whereas one study suggested that patients treated with atorvastatin may be at a lower risk of developing new onset diabetes [14].

The aim of this review was to look systematically into the current literature and carefully collect and analyse results to explore the potential effect of atorvastatin in both causing new onset diabetes and deterioration of glycemic control in patients with known diabetes.

## 2. Methods

### 2.1. Literature Search

This review has adopted the Preferred Reporting Items for Systematic Reviews and Meta-Analyses (PRISMA) guidelines. A systematic search strategy was followed. PubMed database was used to search for publications of interest using as keywords “atorvastatin AND diabetes.” Eligible studies were primary studies of every design (observational studies, cross-sectional, cohort, case studies, case series, clinical trials, etc.) published in English until 30/04/2015 (date of last search). Secondary studies (reviews, letters, and meta-analyses) as well as studies published in languages other than English were excluded. Two reviewers working independently conducted the literature search.

### 2.2. Data Collection and Synthesis

The titles of studies, which were considered for retrieval, were recorded on a form and then were classified on an inclusion and exclusion search diary. All the articles that came up but were irrelevant or were secondary research were excluded. Studies were selected for retrieval after two independent reviewers had collected titles and abstracts identified in electronic searches. The results of the two reviewers were compared by a third independent reviewer, and any differences of opinion were resolved by discussion. The corresponding authors were contacted on account of missing data. The included studies were grouped and presented in Summary Tables featuring key points of each study; the following data were collected: first author surname, study name, year of publication, study design, country, number of total population (percentage of male/female), total population age (Mean-Standard Deviation-Median range), Quantitative results (HR, *p*) of the study findings, diabetes status of the participants, and evidence of association between atorvastatin use and new onset diabetes mellitus or increased risk for worsening of glycemic control. Outcome measures of included studies were organized and then analysed cumulatively. Given the lack of primary data, a narrative form of synthesis was adopted as a way of expressing and synthesizing the results of the eligible studies (i.e., numerical data expressed as weighted means whenever possible). No further statistical analysis was possible.

## 3. Results

**Figure 1:**
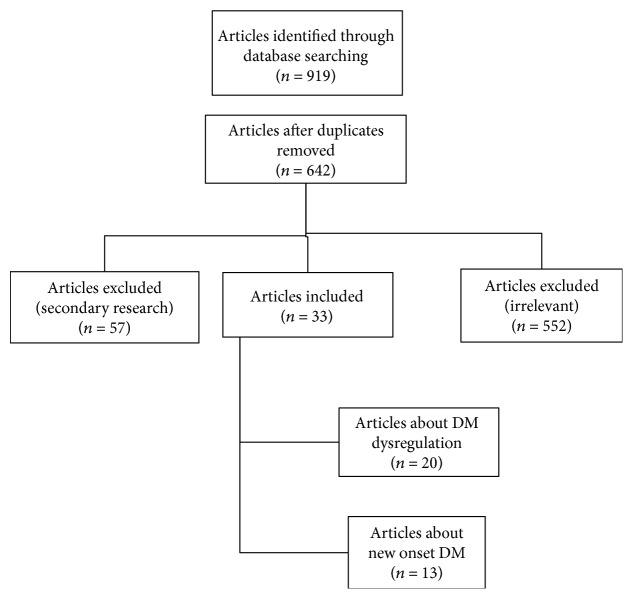
Flowchart of the systematic review process.

In total, 919 articles were identified through database searching, which were reduced to 642 articles after removing duplicates. In all, 33 articles were eligible for the review, following exclusions. Eighteen clinical trials [1–3, 5, 9, 10, 13, 17, 19–28], 14 cohort studies [11, 12, 14–16, 18, 29–36], and one case control study [37] involving a total of 1,951,113 participants were included in the current review. A relevant flow chart was constructed to detail the number of papers retrieved and the steps undertaken (Figure 1).

It should be noted that the primary endpoints of the included studies were cardiovascular disease, LDL-cholesterol levels, HDL-cholesterol levels, or other outcomes such as serum triglyceride levels, apolipoprotein B, serum dehydroepiandrosterone sulfate levels, and C-reactive protein levels. However, all the included studies reported evidence of glycemic control or the incidence of new onset diabetes as secondary endpoints. The proportion of females in the studies ranged between 0% and 100%. More specifically, the total female population was 1,197,855–1,197,955 (approximately 1,197,900) (61.4%). Three studies had only females [15, 25, 37] and 2 studies had only males [28, 34]. In the majority of studies, females were almost as many as males.

The mean average age of the subjects by study (for which age data were available) ranged between 44 and 74.9 years. Thirteen studies included only patients with type 2 diabetes mellitus [2, 3, 10, 11, 17–19, 21, 27–30, 33], while 3 studies included only patients with type 1 diabetes mellitus [13, 20, 24]. The average time since diagnosis of diabetes ranged between 4 and 26 years. The participants had been followed for an average of 4 months to 5 years (weighted average, 3.6 years).

Of note, Koh et al. [9] compared the effect of atorvastatin at doses of 10 mg, 20 mg, 40 mg, and 80 mg. Fasting plasma insulin (mean changes: 25%, 42%, 31%, and 45%) and HbA1c levels (2%, 5%, 5%, and 5%) were increased by atorvastatin 10, 20, 40, and 80 mg when contrast with either baseline (all *p* < 0.05 by paired *t*-test) or placebo (*p* = 0.009 for insulin and *p* = 0.008 for HbA1c by ANOVA). Atorvastatin 10, 20, 40, and 80 mg declined insulin sensitivity (1%, 3%, 3%, and 4%, respectively) when compared with either baseline (*p* = 0.312, *p* = 0.008, *p* < 0.001, and *p* = 0.008, respectively, by paired *t*-test) or placebo (*p* = 0.033 by ANOVA).

Baseline characteristics of the enrolled participants were generally similar between the groups in each study. Information regarding the included studies is presented in Tables 1 and 2.

The mean baseline HbA1c across the studies (weighted average) was 7.2% (56 mmol/mol) in the statin group and 7.3% (57 mmol/mol) in the control group. At the end of the studies, the mean HbA1c was 7.4% (57 mmol/mol) in the statin group and 7.2% (55 mmol/mol) in the control group. The mean baseline fasting glucose across the studies (weighted average) was 7.28 mmol/L in the atorvastatin group and 7.49 mmol/L in the control group. At the end of the studies, the mean fasting glucose was 7.84 mmol/L in the atorvastatin group and 7.20 mmol/L in the control group.

### 3.1. New Onset Diabetes Mellitus

Thirteen articles [1, 12, 14–16, 22, 26, 31, 32, 34–37], 3 of which were clinical trials, examined the association between atorvastatin use and NOD. Patients who used atorvastatin had a higher risk of developing NOD. Also, the results of the majority of studies indicated that the risk of diabetes was dose dependent for atorvastatin. The majority of the articles and all the clinical trials showed that high-dose, compared with lower-dose, atorvastatin increased the risk of NOD. Waters et al. [22] noted that in the TNT clinical trial the HR was 1.10 between those who took atorvastatin 10 mg and 80 mg. Further, the absolute rates of new onset T2DM were 6.40% in the group who took atorvastatin and 6.06% in the placebo group according to the SPARCL clinical trial. The SPARCL clinical trial was a multicenter, double blind, parallel-group, randomized, placebo-controlled trial which randomized patients with prior stroke or transient ischemic attack (TIA) but without known heart disease [38].

However, in one study, the low dose of atorvastatin was related to a small increased risk of NOD (OR: 1.99, 95% CI: 1.00–3.98, *p* value 0.050) [36].

Moreover, statin medication use in postmenopausal women was shown to be associated with an increased risk for DM [15].

### 3.2. Dysregulation of Glycemic Control

Twenty articles examined if deterioration of diabetes mellitus is associated with the use of atorvastatin [2, 3, 5, 9–11, 13, 17–21, 23–25, 27–30, 33], 15 of which were clinical trials. Worsening of glycemic control was examined by measuring parameters related to glucose level such as fasting plasma glucose and HbA1c.

Ten studies showed that there was no significant change in glycemic control in patients treated with atorvastatin [2, 3, 17–19, 24, 27–30].

On the other hand, in 8 studies, atorvastatin use tended to be an independent predictor of increasing HbA1c levels and/or fasting plasma glucose levels (*p* < 0.001) [9–11, 13, 20, 21, 23, 33].

In addition, it was noticed that fasting plasma glucose and HbA1c increased in the group that received higher doses of atorvastatin [5].

The rates of new onset T2DM were higher in groups that took higher dose of atorvastatin. The absolute rates of new onset T2DM were 9.24% in the group who took 80 mg atorvastatin and 8.11% in the group who took 10 mg atorvastatin according to the TNT clinical trial [22]. Among clinical trials, the majority of studies indicated that deregulation of diabetes mellitus (expressed mostly with increased HbA1c levels) was more frequent in the statin group than in the placebo group.

## 4. Discussion

Evidence from randomized clinical trials suggests that the benefits from preventing cardiovascular disease and mortality with statins overweigh the risk of new onset diabetes mellitus [39]. Nevertheless, in patients at low risk for cardiovascular implications, lipid-lowering therapy with statins should be carefully used, and lifestyle changes along with close blood glucose levels monitoring should constitute the first line of treatment [40]. Therefore, it is absolutely necessary for clinical doctors to evaluate the positive and the potential negative effects of statin therapy, taking into consideration the unique characteristics of each patient.

However, according to several studies, the risk of developing NOD varies with different types of statins. Patients who are treated with pravastatin and pitavastatin are in lower risk for adverse effects [41] than those treated with lipophilic statins, such as atorvastatin [11]. Furthermore, the dose of atorvastatin plays an important role. Higher dosage and more intensive treatment are associated with greater incidence of NOD [42]. It should also be noted that older patients, with impaired fasting glucose prior to the use of statins and with other characteristics of the metabolic syndrome, face a greater risk for diabetogenicity [43].

Not many systematic reviews or meta-analyses investigating the association of atorvastatin with NOD and glycemic control dysregulation have been published recently. More specifically, the latest review [44] examining this association, published in 2017, referred only to the correlation between different statins and new onset diabetes mellitus and did not provide detailed data concerning atorvastatin. Also, four previous meta-analyses [45–48] and one review [49] included data concerning all different statins and NOD, but there were no data about statins deteriorating the glycemic control in patients with preexisting diabetes. One meta-analysis [50] and one review [51] underlined only the detrimental effect of atorvastatin, among other statins, on the glycemic control in diabetic patients, unlike our review which is mainly concentrated on the effect of atorvastatin specifically on both NOD and deterioration of the glycemic control.

### 4.1. New Onset Diabetes Mellitus

In this review, we found that there is an association between atorvastatin use and new onset diabetes (NOD). The diabetogenic effect is more significant with high dose of atorvastatin [1, 32, 34, 37], although new data from a most recent cohort study suggest that low-dose atorvastatin (10–20 mg) consists a risk factor for NOD [36], though results were based on a relatively small number of participants (*N* = 818). A recent study investigated whether there is association between statin use and new onset diabetes in postmenopausal women who took part in the Women's Health Initiative. It was revealed that all statins increase the risk of type 2 diabetes mellitus. Particularly, atorvastatin was associated with 61% increased risk of diabetes [15]. One cohort study demonstrated that, in comparison with pravastatin, patients treated with atorvastatin faced a 22% increase in the risk of new onset diabetes [12]. Another analysis, which collated data from 3 different clinical trials (TNT, SPARCL, and IDEAL) on atorvastatin, suggested that atorvastatin at the maximum dose (80 mg) increased the risk for new onset diabetes by 34%, and this was more obvious in the SPARCL trial [22]. Later results from the same clinical trials indicated that the risk for new onset diabetes for patients with 2–4 risk factors was elevated by 24% with high-dose atorvastatin, while there was no diabetogenic effect on patients with 0–1 risk factor [1]. It should also be mentioned that atorvastatin treatment caused dysglycemia (impaired fasting glucose and diabetes mellitus) in a high percentage of renal allograft recipients as indicated by a recent cohort study [16]. However, a retrospective cohort study has shown that atorvastatin had a neutral effect on new onset diabetes, which is dose-response [31], and another study reported that not only does atorvastatin not elevate the risk of diabetes but also may have a protective effect for elderly hypertensive and dyslipidaemic patients [14]. A potential explanation for this may be that the participants had median age of 74.9 years, and there is no record of the dosage of atorvastatin used during the study. No association between atorvastatin and new onset diabetes was demonstrated by one study [26], but this may be attributed to the fact that the lowest dose of atorvastatin (10 mg) was used for this study.

### 4.2. Dysregulation of Glycemic Control

As far as glycemic control is concerned, this review has shown that high dose of atorvastatin is associated with the deterioration and worsening of glucose homeostasis [13, 21, 23, 33]. A clinical trial published in 2010 reported that atorvastatin 10 mg compared to atorvastatin 80 mg increased fasting plasma glucose (FPG) by 25% and 45%, respectively, and HbA1c by 2% and 5%, respectively [9]. The influence of high-dose atorvastatin on glycemic control was also demonstrated by 2 different studies, which reported a significant increase (0.3%) of HbA1c compared to baseline along with no alteration in FPG levels [10, 20].

No significant changes in glycemic control between the atorvastatin group and the control group were found by a clinical trial in China [19], but this was a low-quality study (follow-up of 6 months in population of 80 patients and the exact dose of atorvastatin used was not reported). Furthermore, a mediocre increase (0.06%) of HbA1c was documented for patients treated with atorvastatin compared to the control group according to a recent study; however, no data regarding the dosage of atorvastatin were available [30]. Another study that has also shown no difference in the glycemic control is a clinical trial from Greece, which included only 79 participants who were treated with a low dose (10 mg) of atorvastatin [18]. A neutral effect of atorvastatin 10 mg was also reported by 3 more studies [3, 27, 28]. Finally, 2 clinical studies which used atorvastatin 20 mg [2, 25] and one with atorvastatin groups of 10-20-40 mg, respectively [17, 25], showed no association.

A positive effect on HbA1c levels was suggested by one study investigating the effects of atorvastatin in renal function of patients with diabetes [29]. This retrospective cohort study showed that HbA1c was significantly decreased at 1, 2, and 3 years. There was no information about the dosage of atorvastatin. In a different trial, a nonsignificant decrease of HbA1c was reported in patients treated with atorvastatin high dose (80 mg) compared to placebo [24].

### 4.3. Mechanisms

Lipid-lowering drugs intervene with glucose control and insulin sensitivity in many different ways. Mainly, HbA1c and FPG are affected. Although the precise underlying pathogenetic mechanisms that lead to the development of diabetes are not yet scientifically proven, there are some studies which suggest that the intervention of statins in the Mevalonate path and hence in the isoprenoids' synthesis is connected with the deterioration of glycemic control [9]. Statin treatment also results in the downregulation of glucose transporter 4 (GLUT4) in adipocytes, which causes insulin resistance [52]. Furthermore, statins decrease insulin secretion via the decrease of glucose-dependent intracellular calcium concentration [53] and via the inhibition of ubiquinone (CoQ10), which leads to reduction of ATP in pancreatic *β*-cells [54]. These effects are more intense with lipophilic statins like atorvastatin than with hydrophilic statins like pravastatin [55]. Other plausible mechanisms, including genetic factors, have also been explored but with no concrete evidence yet.

### 4.4. Drugs That May Have a Metabolic Effect on Insulin Resistance and on the Development of Diabetes

Several classes of antidiabetic drugs are available. Each category has a distinct pathophysiological mechanism of action and consequently a different effect regarding insulin resistance and *β*-cell pancreatic function.

Indicatively, metformin inhibits hepatic gluconeogenesis and also improves insulin sensitivity via activation of AMP-activated protein kinase (AMPK) signalling and reducing cAMP (cyclic adenosine monophosphate) levels. Moreover, metformin is associated with microbiome modification of the gastrointestinal tract and entails an increase of incretin (glucagon-like peptide-1, GLP-1) secretion and glucose utilization [56].

Pioglitazone binds and activates the peroxisome proliferator-activated receptor (PPAR) *γ* leading to metabolic changes concerning carbohydrate and lipid metabolism. It is associated with an increase in tissue sensitivity to insulin and subsequently an enhanced glucose uptake in the skeletal muscle and adipose tissue. Also, it causes a reduction in hepatic glucose production and an increase in hepatic glucose uptake. It may stimulate *β*-cell insulin production and may have beneficial effects both on endothelial and pancreatic *β*-cells [57–59].

Sulfonylureas stimulate the endogenous secretion of insulin from pancreatic *β*-cells by inhibiting the ATP-sensitive K-channels. Therefore, sulfonylureas exert their effects only when residual *β*-cells exist [60, 61].

Reduced incretin levels may play a part in the pathogenesis of T2DM. Incretin-based therapies, dipeptidyl peptidase-4 (DPP-4) inhibitors, and GLP-1 receptor agonists (GLP-1RAs) affect glucose control via pleiotropic mechanisms and play a significant role in glucose homeostasis.

GLP-1RAs enhance glucose-dependent insulin secretion, delay gastric emptying, and reduce food intake and postprandial glucagon secretion [62].

DPP4 inhibitors delay the inactivation of incretin hormones, also resulting in increased insulin synthesis and decreased glucagon levels in a glucose-dependent manner [63].

According to clinical and preclinical study results, incretin-based therapies may have a beneficial effect on hepatic steatosis and steatohepatitis, inhibit intestinal lipoprotein production, enhance *β*-cell function, and produce multiple biological actions in peripheral tissues [63, 64].

On the other hand, various nondiabetic drugs seem to play a crucial role in insulin sensitivity and endothelial dysfunction [65]. Several experimental evidence suggests that overactivity of renin-angiotensin-aldosterone system (RAAS), namely angiotensin II, interferes with insulin resistance and glucose metabolism [66]. Thus, drugs with RAAS blockade activity may interact with the skeletal muscle, adipose tissue, or pancreas contributing to altered glucose metabolism and insulin sensitivity.

It has been shown that angiotensin II interferes with insulin metabolic signalling, induces insulin resistance, and impairs insulin-stimulated glucose disposal. It inhibits insulin receptor substrates 1 and 2 (IRS-1, IRS-2), enhances serine phosphorylation affecting the PI3K (phosphoinositide 3-kinase) pathway, impairs the insulin-mediated vasodilation, and also reduces the ability of IRS-1 to interact with the activated insulin receptor [67, 68].

It has also been proposed that angiotensin II is associated with the functional impairment of pancreatic *β*-cells through inflammation, attenuation of islet fibrosis, and oxidative damage of the pancreas. The metabolic stress, as well as the dedifferentiated status of *β*-cells induced by angiotensin II, has also been associated with pancreatic *β*-cell failure and the potential progressive development of T2DM [69–71].

Angiotensin II receptor blockers (ARBs) are drugs that block the action of angiotensin II by selectively inhibiting its binding to angiotensin II receptors on the muscles surrounding blood vessels.

According to some studies, ARB medications improved insulin sensitivity [72–74] while others showed an enhancement in the early phase of insulin secretion, a significant effect possibly attributed to the recovery of pancreatic *β*-cell function [75, 76].

ACE (angiotensin-converting-enzyme) inhibitors are also involved in the renin-angiotensin axis. It has been proposed that ACE inhibitors promote glycemia and glucose tolerance probably though preservation of *β*-cell function [77], improvement of insulin sensitivity (via activation of bradykinin-nitric oxide pathway) [78], anti-inflammatory processes [79], and multiple other underlying mechanisms [80, 81].

It is evident that apart from their antihypertensive effect, both ARBs and ACE inhibitors may exert beneficial effects on lipid and carbohydrate metabolism and insulin resistance [66, 82, 83], which may explain the possible protective role of these medications partially [84–86]. Furthermore, existing evidence demonstrates a potential protective role of RAAS inhibitors in new onset of T2DM [86–88].

Fibrates are a class of hypolipidemic agents that exert their effects through activation of PPARs, namely PPAR*α*, which modulate carbohydrate and lipid metabolism and adipose tissue differentiation. According to several studies, including patients with T2DM, fibrates and especially fenofibrate improved lipidemic parameters, insulin resistance, and glycemic control [89–91]. Moreover, a further investigation is proposed to examine the potential protective role of fibrates in preventing T2DM [92].

Additionally, other lipid-lowering drugs such as ANGPTL3 antisense oligonucleotides are also associated with an improvement of insulin sensitivity [93].

Taking into account all the aforementioned, it seems essential to clarify the underlying pathophysiologic mechanisms of action for each drug, their impact on insulin resistance and on the overall glucose homeostasis especially in patients with T2DM or in patients at high risk of NOD who also receive statin therapy [65].

### 4.5. Strengths and Limitations

This systematic review includes approximately 2,000,000 participants in total from all studies, contributing to a large cohort. In addition, a comprehensive literature search was followed, as well as bias protection methods such as three independent reviewers.

Of note, our data were extracted from more recent clinical studies, the latest of which conducted in 2015, in contrast to aforementioned review studies. Two reviews [41, 94], with clinical trials until 2009 and 2013, respectively, found no association between atorvastatin and NOD as well as atorvastatin and deterioration of the glycemic control. The review by Kostapanos et al. [94] included a very small number of subjects and mainly low doses of atorvastatin, while the review by Naci et al. [41] investigated only the incidence of NOD in trials that were not designed for this purpose and had no information about the doses of atorvastatin used.

However, the limitations of the review should be acknowledged. Firstly, the medical status of each participant (e.g., coexisting diseases) and the concomitant medications (especially the glucose-lowering agents or other drugs with metabolic impact, as already mentioned) were not taken into account, since data were not consistently available. Furthermore, only clinical studies published in PubMed were included, thus results from nonindexed trials are missing. All studies not published in the English language were excluded. Further, about a third (13 of 33) of the included studies used observational designs. Finally, most of the studies were not designed to investigate the association between atorvastatin and new onset diabetes or dysregulation of existing diabetes mellitus, but their primary designation purpose was different, contributing to the heterogeneity of the results.

## 5. Conclusions

Our findings suggest that there is association between atorvastatin treatment and new onset diabetes mellitus. It was also demonstrated that atorvastatin causes worsening of glycemic control in patients with known diabetes but only in maximum dose and not in lower doses. Nevertheless, serious consideration needs to be placed on the cost-benefit ratio and the potential importance of these adverse effects of atorvastatin when compared to the scientifically and clinically observed beneficial effects of statins on cardiovascular risk. Given the wide availability of statins and relevant databases, more studies using routine clinical data are required to be conducted, on wider homogeneous populations of participants and with larger periods of follow-up, in order to clarify the real association between statin therapy and the development of new onset diabetes mellitus. Moreover, the impact of other coadministrated drugs on insulin resistance and glucose homeostasis should be taken into deep consideration.

Hence, our review underlines the existence of a significant yet not so thoroughly investigated issue, which is the development of NOD and also the potential deterioration of the glycemic control by atorvastatin in patients with diabetes.

## Figures and Tables

**Table 1:** Atorvastatin use and new onset diabetes mellitus.

Study author, year	Design	Study location	Total population (% F)	Total population: age, mean (SD), median (range/IQR)	Duration	Comparison groups	Risk of developing NOD (statin users vs nonstatin users)
Waters et al., 2011 [4, 22, 38, 95]	3 clinical trials	TNT: worldwide	TNT: 7595 (17%)	TNT: 60.6 (8.9), NA	TNT: 4.9 years	TNT: atorvastatin 10 mg and atorvastatin 80 mg	TNT: HR: 1.10 (0.94–1.29), *p* = 0.22
		IDEAL: northern Europe	IDEAL: 7461 (19%)	IDEAL: 61.5 (9.5), NA	IDEAL: 4.8 years	IDEAL: atorvastatin 80 mg and simvastatin 20 mg	IDEAL: HR: 1.19, (0.99–1.44), *p* = 0.072
		SPARCL: worldwide	SPARCL: 3803 (41%)	SPARCL: 62.5 (11.6), NA	SPARCL: 4.9 years	SPARCL: nonstatin users and atorvastatin 80 mg	SPARCL: HR: 1.34, (1.05–1.71), *p* = 0.018

Waters et al., 2013 [1, 4, 95]	2 clinical trials	TNT: worldwide	15,056 (18%)	61.1 (9.2), NA	TNT: 4.9 years	TNT: atorvastatin 10 mg and atorvastatin 80 mg	0–1 risk factors: HR: 0.97 (0.77–1.22)
		IDEAL: northern Europe	[TNT: 7595, IDEAL: 7461]		IDEAL: 4.8 years	IDEAL: atorvastatin 80 mg and simvastatin 20 to 40 mg	2 to 4 risk factors: HR: 1.24 (1.08–1.42), *p* = 0.0027

Sever et al., 2003 [26]	Clinical trial	UK, Ireland, Nordic countries	10,305 (19%)	63 (NA), NA (40–79)	3.3 years	Atorvastatin 10 mg and nonstatin users	HR: 1.15 (0.91–1.44), *p* = 0.2493

Chen et al., 2013 [37]	Case control	Taiwan	11,715 (100%)	NA, NA	2 years	Atorvastatin users and nonstatin users	Adj. OR: 2.80 (1.74–4.49), *p* < 0.001

Ma et al., 2012 [14]	Retrospective cohort study	Taiwan	15,637 (NA)	74.9 (6.3), NA	5.5 years	Atorvastatin users and nonstatin users	Adj. HR: 0.77 (0.72–0.83), *p* < 0.0001

Ma et al., 2012 [31]	Retrospective cohort study	Taiwan	16,027 (54%)	59.9 (18.7), NA (20–84)	3.5 years	Atorvastatin users and nonstatin users	Users vs non users: Adj. HR: 1.29 (1.16–1.44), *p* < 0.0001
							Among users: Adj. HR: 1.15 (0.96–1.35), *p* = 0.5465

Carter et al., 2013 [12]	Retrospective cohort study	Canada	471,250 (54%)	NA, 73 (69–78)	12.5 years	Atorvastatin users and pravastatin users	All users: Adj. HR: 1.22 (1.15–1.29) Primary prevention users: 1.20 (1.10–1.30) Secondary prevention users: 1.25 (1.16–1.34)

Cho et al., 2015 [35]	Retrospective cohort study	Korea	3680 (52.01%)	NA, NA	62.6 (15.3) months	Atorvastatin users and simvastatin users	Adj. HR = 1.52(0.72–3.21), *p* = 0.268

Zaharan et al., 2013 [32]	Retrospective cohort study	Ireland	1,235,671 (61%)	NA, NA	8.5 years	Atorvastatin users and nonstatin users	Adj. HR: 1.25 (1.21–1.28), *p* < 0.0001

Choe et al., 2014 [16]	Cohort study	Korea	394 (42%)	NA, NA	5 years	Atorvastatin users and nonstatin users	Adj. HR: 3.76 (2.22–6.40), *p* = 0.001
Culver et al., 2012 [15]	Cohort study	USA	153,840 (100%)	63.17 (7.25) NA (50–79)	*NA*	Atorvastatin users and nonstatin users	Adj. HR: 1.61 (1.26–2.06)

Cederberg et al., 2015 [34]	Cohort study	Finland	8749 (0%)	57 (7) 57 (45–73)	5.9 years	Atorvastatin users and nonstatin users	Adj. HR: 1.21 (1.04–1.40)
						Atorvastatin users (20 or 40 mg) and nonstatin users	HR: 1.37 (1.14–1.65)

Park et al., 2015 [36]	Cohort study	Korea	Initial: 3566 (49.41%), after PSM adjustment: 818 (49.14%)	NA, NA	3 years	Atorvastatin users (10 or 20 mg) and nonstatin users	OR: 1.99 (1.00–3.98), *p* = 0.050

Variables are expressed as absolute numbers, percentages, mean ± SD, and median (IQR). NOD: new onset diabetes mellitus; NA: not available; PSM: propensity score matching analysis; Adj. HR: adjusted hazard ratio.

**Table 2:** Atorvastatin use and dysregulation of diabetes mellitus.

Study author, year	Design	Study location	Total population (% F)	Total population: age, mean (SD), median (range/IQR)	Comparison groups	DM type	Duration of study	Quantitative results related to atorvastatin
Tam et al., 2010 [19]	Clinical trial	China	80 (NA)	NA, NA	Atorvastatin and placebo	T2DM	6 months	FPG (mmol/L) (vs baseline) Placebo: 7.95 ± 1.99 → 7.56 ± 2.06, ns Atorvastatin: 7.91 ± 2.10 → 8.03 ± 2.48, ns
								HbA1c (%) [mmol/mol] (vs baseline) Placebo: 8.0 ± 1.1 [64 ± 11] → 8.0 ± 1.2 [64 ± 10], ns Atorvastatin: 7.8 ± 1.2 [62 ± 10] → 8.2 ± 1.4 [66 ± 8], ns
								Placebo vs atorvastatin: ns

Mandosi et al., 2010 [2]	Clinical trial	Italy	22 (23%)	60.8 (7.1), NA	All atorvastatin 20 mg users	T2DM	8 weeks	From baseline HbA1c (%) [mmol/mol]: 7.6 ± 1.1 [60 ± 11] → 7.6 ± 0.9 [60 ± 14], *p* = 0.52 FPG (mmol/L): 8.6 ± 2.2 → 9.1 ± 1.9, *p* = 0.36

Koh et al., 2010 [9]	Clinical trial	Korea	213 (50%)	NA, NA	Atorvastatin 10 mg, 20 mg, 40 mg, 80 mg, and placebo	T2DM & without diabetes	2 months	HbA1c (%) [mmol/mol] Placebo: 5.8 ± 0.5 [40 ± 18] → 5.8 ± 0.6 [40 ± 17], ns 10 mg: 5.8 ± 0.6 [40 ± 17] → 6.0 ± 0.6 [42 ± 17] (*p* < 0.001 vs baseline) 20 mg: 5.9 ± 0.8 [41 ± 15] → 6.2 ± 0.9 [44 ± 14] (*p* < 0.001 vs baseline, *p* < 0.05 vs placebo) 40 mg: 6.1 ± 0.8 [43 ± 15] → 6.4 ± 1.0 [46 ± 13] (*p* < 0.01 vs baseline, *p* < 0.05 vs placebo) 80 mg: 6.1 ± 0.8 [43 ± 15] → 6.4 ± 1.1 [46 ± 11] (*p* < 0.05 vs baseline, *p* < 0.05 vs placebo)
								Global ANOVA: *p* = 0.008

Tehrani et al., 2010 [20]	Clinical trial	Sweden	20 (NA)	NA 44 (39–61)	Atorvastatin 80 mg and placebo	T1DM	2 months	HbA1c (%) [mmol/mol] Placebo: 7.6 ± 0.9 [60 ± 14] → 7.3 ± 0.8 [56 ± 15] Atorva 80 mg: 7.5 ± 0.9 [58 ± 14] → 7.8 ± 1.1 [62 ± 11], *p* < 0.01
								Atorva vs placebo: *p* < 0.001

Rutter et al., 2011 [21]	Clinical trial	UK	119 (17%)	64 (10) NA	Atorvastatin 80 mg and atorvastatin 10 mg	T2DM	2.1 years	HbA1c (%) [mmol/mol] (vs baseline) Atorva 10 mg vs 80 mg: 0.3 [2.4] (0.1–0.5) [0.4–4.3], *p* = 0.017
Tehrani et al., 2013 [13]	Clinical trial	Sweden	20 (50%)	NA 44 (39–61)	Atorvastatin 80 mg and placebo	T1DM	2 months	HbA1c (%) [mmol/mol] Atorvastatin treatment period: 7.5 ± 0.9 [58 ± 14] → 7.8 ± 1.1 [62 ± 11], *p* = 0.008 Atorvastatin vs placebo: *p* < 0.001

Grigoropoulou et al., 2014 [18]	Clinical trial	Greece	79 (61%)	NA NA (45–75)	Atorvastatin 10 mg and control	T2DM	12 months	HbA1c (%) [mmol/mol], (0 → 3mo → 6mo → 12mo) Atorvastatin: 6.7 ± 0.8 [50 ± 15] → 6.7 ± 0.7 [50 ± 16] → 6.8 ± 0.8 [51 ± 15] → 6.9 ± 0.7 [52 ± 16], *p* = 0.09 Control: 7.0 ± 0.7 [53 ± 16] → 6.8 ± 0.6 [51 ± 17] → 6.9 ± 0.7 [52 ± 16] → 7.0 ± 0.7 [53 ± 16], *p* = 0.06 Atorvastatin vs control: *p* = 0.26
								FPG (mg/dL), (0 → 3mo → 6mo → 12mo) Atorvastatin: 141.2 ± 33.3 → 138.1 ± 27.5 → 136.2 ± 29.8 → 138.3 ± 29.8, *p* = 0.76 Control: 153.9 ± 50.6 → 142.1 ± 54.3 → 142.7 ± 42.2 → 145.6 ± 35.5, *p* = 0.36 Atorvastatin vs control: *p* = 0.21

Thongtang et al., 2011 [23]	Clinical trial	USA	272 (49%)	NA, NA	Atorvastatin 80 mg, rosuvastatin 40 mg	T2DM & without diabetes	6 weeks	HbA1c (%) [mmol/mol] Mean differences vs baseline: Atorvastatin: +0.1 [22] (−2.6–9.3) [−52–78]

Martin et al., 2011 [24]	Clinical trial	Germany	89 (40%)	30 (NA), NA (18–39)	Atorvastatin 80 mg and placebo	T1DM	18 months	HbA1c (%) [mmol/mol] (baseline → 6mo → 18mo) Atorvastatin vs baseline: 7.8 [62] → 6.6 [49] → 6.8 [51], *p* < 0.001
								Atorvastatin vs placebo: ns

Simsek et al., 2012 [10]	Clinical trial	Netherlands	263 (54%)	60 (10), NA	Atorvastatin and rosuvastatin	T2DM	24 weeks	HbA1c (%) [mmol/mol] 18w vs baseline: Atorva 80 mg: 7.4 ± 1.0 [57 ± 13] → 7.7 ± 1.3 [61 ± 9], *p* = 0.003 HbA1c 6w vs baseline: Atorva 20 mg: 7.4 ± 1.0 [57 ± 13] → 7.5 ± 1.1 [58 ± 11], ns
								Mean FPG (mmol/L) 18w vs baseline: Atorva 80 mg: 8.7 ± 2.4 → 9.0 ± 3.0, ns Mean FPG (mmol/L) 6w vs baseline: Atorva 20 mg: 8.7 ± 2.4 → 9.5 ± 3.0, *p* = 0.002

Puurunen et al., 2013 [25]	Clinical trial	Finland	28 (100%)	NA NA (29–50)	Atorvastatin 20 mg and placebo	Without diabetes	6 months	FPG, mmol/L (0 → 3mo → 6mo) Atorva: 5.5 ± 0.3 → 5.5 ± 0.4 → 5.5 ± 0.4, *p* = 0.763 Placebo: 5.3 ± 0.3 → 5.3 ± 0.3 → 5.0 ± 0.5, *p* = 0.076 Atorva vs placebo (6mo): *p* = 0.007

Chu et al., 2008 [17]	Clinical trial	Taiwan	29 (50%)	60.0 (2.2) NA (18–80)	Atorvastatin 10 mg, 20 mg, 40 mg	T2DM	12 weeks	HbA1c (%) [mmol/mol] 10 mg: 7.6 ± 0.4 [60 ± 19] → 7.4 ± 0.3 [57 ± 20], ns
								20 mg: 8.2 ± 0.3 [66 ± 20] → 8.0 ± 0.3 [64 ± 20], ns
								40 mg: 8.3 ± 0.3 [67 ± 20] → 8.7 ± 0.5 [72 ± 18], ns

Goyal et al., 2014 [27]	Clinical trial	India	43 (37.21%)	NA, NA	Atorvastatin 10 mg and placebo	T2DM	12 weeks	HbA1c, (%) [mmol/mol] (vs baseline) Atorva: 7.6 ± 0.9 [60 ± 14] → 7.6 ± 1.4 [60 ± 8], *p* = 0.92 Placebo: 7.9 ± 0.9 [63 ± 14] → 7.4 ± 1.8 [57 ± 4], *p* = 0.25
								FPG (mmol/L) (vs baseline) Atorva: 7.44 ± 1.52 → 7.99 ± 2.18, *p* = 0.18 Placebo: 8.18 ± 1.86 → 8.16 ± 3.28, *p* = 0.95

Ogawa et al., 2014 [3]	Clinical trial	Japan	1018 (54.22%)	66.4 (11.1) NA	Atorvastatin 10 mg and rosuvastatin 5 mg	T2DM	12 months	HbA1c (%) [mmol/mol] (0 → 6mo → 12mo) Atorva: 6.4 [46] → 6.5 [48] → 6.5 [48], ns
								Glu (mg/dL) (0 → 6mo → 12mo) Atorva: 118.8 → 126.0 → 122.8, *p* < 0.001
Sadeghi et al., 2014 [5]	Clinical trial	Iran	140 (55%)	NA, NA	Atorvastatin 40 mg and atorvastatin 20 mg	Without diabetes	3 months	HbA1c (%) [mmol/mol] (0 → 3mo): Atorva 40 mg: 5.5 ± 0.6 [37 ± 17] → 5.9 ± 0.6 [41 ± 17], *p* < 0.001 Atorva 20 mg: 5.5 ± 0.7 [37 ± 16] → 5.5 ± 0.6 [37 ± 17], *p* = 0.442 Atorva 40 mg vs 20 mg: *p* < 0.001
								FPG (mg/dL) (0 → 3mo): Atorva 40 mg: 85.67 ± 13.59 → 99.86 ± 16.22, *p* < 0.001 Atorva 20 mg: 84.63 ± 26.26 → 85.21 ± 14.19, *p* = 0.656 Atorva 40 mg vs 20 mg: *p* < 0.001

Black et al., 2014 [28]	Clinical trial	UK	13 (0%)	61.3 (2.5), NA (35–70)	Atorvastatin 10 mg and fenofibrate 267 mg	T2DM	12 weeks	HbA1c (%) [mmol/mol] (0 → 12w) Atorva 10 mg: 7.0 ± 0.2 [53 ± 21] → 7.1 ± 0.3 [54 ± 20], ns Fenofibrate 267 mg: 7.1 ± 0.2 [54 ± 21] → 7.0 ± 0.3 [53 ± 20], ns Atorva10 mg vs fenofibrate 267 mg: *p* = 0.52
								FPG (mmol/L) (0 → 12w) Atorva 10 mg: 7.6 ± 0.3 → 8.4 ± 0.5, ns Fenofibrate 267 mg: 8.1 ± 0.5 → 7.7 ± 0.5, ns Atorva 10 mg vs fenofibrate 267 mg: *p* = 0.07

Tanaka, 2011 [29]	Study 1: prospective cohort study	Japan	114 (49%)	NA, NA	Study 1: (statin-naïve and other statin users) all switched to atorvastatin 10 mg	T2DM	Study 1: 3 months	HbA1c (%) [mmol/mol] Study 1 (vs baseline) Statin-naïve: 7.6 ± 1.1 [60 ± 11] → 7.5 ± 0.9 [58 ± 14], ns Statin users: 7.1 ± 1.1 [54 ± 11] → 7.1 ± 1.0 [54 ± 13], ns
	Study 2: retrospective cohort study				Study 2: all atorvastatin users		Study 2: 3 years	Study 2 (vs baseline) Baseline: 7.8 ± 1.5 [62 ± 7] → 1y: 7.6 ± 1.4 [60 ± 8], *p* < 0.05 → 2y: 7.4 ± 1.5 [57 ± 7], *p* < 0.01 → 3y: 7.5 ± 1.5 [58 ± 7], *p* < 0.01
Yamakawa et al., 2008 [11]	Retrospective cohort study	Japan	279 (54%)	NA, NA	Atorvastatin 10 mg Pitavastatin 2 mg Pravastatin 10 mg	T2DM	3 months	HbA1c (%) [mmol/mol] Atorva: 7.0 ± 1.1 [53 ± 11] → 7.4 ± 1.2 [57 ± 10], *p* < 0.001
								Glu (mg/dL) Atorva: 147 ± 51 → 176 ± 69, *p* < 0.001.

Takano et al., 2006 [33]	Retrospective cohort study	Japan	154 (56%)	NA, NA	Atorvastatin 10 mg, pravastatin 10 mg	T2DM	3 months	HbA1c (%) [mmol/mol] Atorva: 6.8 ± 0.9 [51 ± 14] → 7.2 ± 1.1 [55 ± 11], *p* < 0.001
								Glu (mg/dL) Atorva: 147 ± 50 → 177 ± 70, *p* < 0.001

Shinozaki et al., 2012 [30]	Cohort study	Japan	1173 (54%)	NA, NA (65–84)	Atorvastatin and atorvastatin-untreated	T2DM	6 years	HbA1c (%) (vs baseline) Atorvastatin treatment period: 0.06% (−0.08%–0.21%, *p* = 0.38) Atorvastatin vs untreated: 1.69 (0.42–6.84)

Variables are expressed as absolute numbers, percentages, mean ± SD, and median (IQR). NA: not available; NS: no significance; DM: diabetes mellitus; T2DM: type 2 diabetes mellitus; T1DM: type 1 diabetes mellitus; FPG: fasting plasma glucose; HbA1c: haemoglobin A1c; Glu: glucose; ANOVA: analysis of variance; Mo: months; W: weeks.

## Abbreviations

NOD: New onset diabetes mellitus
HMG-CoA: 3-Hydroxy-3-methylglutaryl coenzyme A
LDL: Low-density lipoprotein cholesterol
HDL: High-density lipoprotein cholesterol
HR: Hazard ratio
SD: Standard deviation
IQR: Interquartile range
OR: Odds ratio
NA: Not available
NS: No significance
DM: Diabetes mellitus
T2DM: Type 2 diabetes mellitus
T1DM: Type 1 diabetes mellitus
FPG: Fasting plasma glucose
HbA1c: Haemoglobin A1c
Glu: Glucose
ANOVA: Analysis of variance
Y: Years
Mo: Months
W: Weeks
PSM: Propensity score matching analysis
ATORVA: Atorvastatin
ADA: American Diabetes Association
PRISMA: Preferred reporting items for systematic reviews and meta-analyses
ACC/AHA: American College of Cardiology and the American Heart Association
AMPK: AMP-activated protein kinase
cAMP: Cyclic adenosine monophosphate
GLP-1: Glucagon-like peptide-1
GLP-1RAs: GLP-1 receptor agonists
DPP-4: Dipeptidyl peptidase-4
RAAS: Renin-angiotensin-aldosterone system
IRS-1: Insulin receptor substrates 1
IRS-2: Insulin receptor substrates 2
ARBs: Angiotensin II receptor blockers
ACE: Angiotensin-converting-enzyme
PPARs: Peroxisome proliferator-activated receptors.

## Additional Points

*Key points*. Atorvastatin may cause dysregulation of glycemic control in patients with diabetes. Association between atorvastatin treatment and new onset diabetes mellitus. Deterioration of glycemic control is increased after high-dose atorvastatin therapy. Association of new onset diabetes mellitus with high-dose atorvastatin therapy. More studies are required to clarify the underlying mechanisms and the real association between statin therapy and the development of new onset diabetes mellitus.
